# Sexually antagonistic selection during parental care is not generated by a testosterone-related intralocus sexual conflict–insights from full-sib comparisons

**DOI:** 10.1038/srep17715

**Published:** 2015-12-02

**Authors:** Arne Iserbyt, Marcel Eens, Wendt Müller

**Affiliations:** 1 Department of Biology–Ethology, University of Antwerp, Universiteitsplein 1, B-2610 Wilrijk, Belgium

## Abstract

The evolution of shared male and female traits can be hampered if selection favours sex-specific optima. However, such genomic conflicts can be resolved when independent male and female mechanisms evolve. The existence, extent and consequences of conflict and/or conflict resolution are currently debated. Endocrinological traits like plasma testosterone (T) are suitable test cases, given their important role in mediating correlated traits, plus their opposing sex-specific fitness effects. We compared full-sibling (brother/sister) captive canaries to test for (1) sexually antagonistic selection characterized by contrasting fitness patterns within pairs of relatives, (2) intersexual genetic correlation of plasma T (*h^²^* = 0.41  ±  0.31) and (3) intralocus sexual conflict over T levels featured by distinct sex-specific fitness optima. We found potential for sexually antagonistic selection, since high fledgling mass was reached by either brothers or sisters, but not by both. We report a positive intersexual correlation for T, as a requirement for intralocus sexual conflict. However, high levels of T were associated with increased female and decreased male fitness (fledgling mass), which contrasts our expectations and challenges the hypothesis of intralocus sexual conflict driven by T. We hypothesize that behavioural and physiological trade-offs differ between sexes when raising offspring, driving T levels towards a state of monomorphism.

The expression of complex vocalizations, brightly colored structures, immunocompetence against pathogens and armaments used in the battle for territories or mates are just snapshots among text book examples that represent the variety of physiological, morphological and behavioural traits mediated by the gonadal steroid hormone testosterone (T) in vertebrates. Variation in the expression of such traits is often closely related to male fitness, so it is not surprising that T has been regarded as the male hormone for nearly a century^1^,^2^. However, females are also known to circulate significant levels of androgens, to possess androgen receptors, as well as androgen metabolizing enzymes^3^. In fact it recently became clear that quantitative changes in individual hormone levels can result in as dramatic phenotypic variability in females as in males^4^,^5^. However despite intense research over the past decades^6^,^7^,^8^, a current debate exists about the extent to which different optimal levels of functional T in both sexes constrain male and female evolution^9^,^10^,^11^,^12^.

Independent evolution of both sexes can from the quantitative genetic point of view be constrained when trait expression is regulated by the same set of genes in males and females^13^. Thus intersexual genetic correlations may cause a fundamental evolutionary problem when selection favours sex-specific phenotypic optima^14^,^15^,^16^. This concept is known as sexually antagonistic selection driven by an intralocus sexual conflict and forms one of the most important genomic conflicts^7^. Evidence for such intralocus sexual conflicts exists for a range of different traits, related to morphology^17^,^18^, locomotory activity^19^ or immune defense^20^. In case of testosterone, increased levels of plasma T in males are typically associated with overall increased lifetime reproductive success, because of enhanced competitive abilities in the context of intra-and intersexual selection^21^. Given this, directional selection for high T levels is expected until the benefits are maximized against the costs involved like immune suppression^22^, increased oxidative stress^23^ or lowered parental care^24^. Despite lower profiles compared to males, T is physiologically meaningful in females too, for example for the development of muscle mass and bone structures, the maintenance of motor neurons^3^, and the transfer of androgens to the offspring^25^. However females also suffer from elevated T levels through lowered reproduction, for example due to reduced nest building behaviour, delayed or inhibited egg laying, decreased brooding or food provisioning^5^,^26^,^27^,^28^. If directional selection acts in opposite directions for males and females and if T is regulated by the same genes, an intersexual genetic correlation for the production of T would impose significant constraints to reach sex-specific fitness optima and thus outlining an intralocus sexual conflict.

However, genetic mechanisms such as sex linkage and sex-specific gene regulation may evolve that decouple developmental pathways for a given trait in males and females^14^,^15^,^16^,^29^. Such processes may erode intersexual correlations, permitting the phenotypes of males and females to diverge toward their own sex-specific optima, resolving an intralocus sexual conflict. Sexual dimorphism is probably the most common outcome of such resolved conflicts. If this is the case for genes involved in T production and the associated receptor proteins, then major sex-specific consequences are expected for the evolution of correlated morphological, physiological and behavioural traits^30^,^31^.

The above sketches the two most extreme views, but in reality, the expression of male and female traits is most likely characterized along a continuum of both correlated responses to selection on the opposite sex and independent sex-specific mechanisms^12^. Indeed, both views should be integrated if we want to understand the evolution of traits shared by both sexes^14^. Despite increased research interest on the adaptive significance of male and female plasma T levels, the question addressed ten years ago by Ketterson and colleagues^4^ still remains; whether female T is a mediator of adaptive traits, a constraint on sexual dimorphism or both. Yet assessing whether T levels in females are adaptive or not, requires knowledge about whether this trait is genetically correlated between sexes. But such fundamental information about the heritability of plasma T levels, especially in birds^32^, is still extremely limited.

In the current study, we make use of a pedigreed captive canary (*Serinus canaria*) population and compare full-sib brothers and sisters in reproductive activities and plasma T levels, close to the peak of T production. That is during ovulation in females and territorial establishment prior to mating in males^4^,^33^. These peak plasma T levels have been shown to be highly repeatable^34^ and easily allows to standardize timing of blood sampling within and across studies. Our aims are threefold: First, we compare plasma T levels of brothers and sisters to explore the existence of an intersexual correlation and to estimate broad-sense heritability of plasma T levels. Second, we assess the existence of sexually antagonistic fitness variation^35^,^36^ by comparing a fitness estimate (i.e. fledgling body mass) between siblings. Third, we test the existence of an intralocus sexual conflict over plasma T levels by investigating whether testosterone affects male and female fitness differently. Specifically, we expect an increase of male fitness and a decrease of female fitness with increasing plasma T levels. Together, this would reflect an intralocus sexual conflict that constrains the evolution of male and female T levels via an intersexual genetic correlation.

## Results

### Intersexual correlation of plasma T

**Figure 1:**
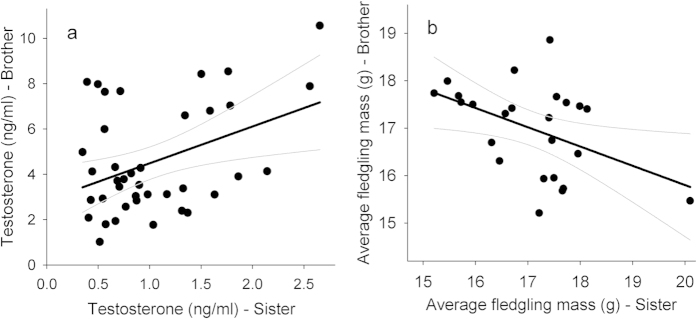
Full-sibling comparisons. Brothers and sisters are compared in (**a**) plasma testosterone levels, (**b**) parental fitness (estimated as average fledgling body mass of the four unrelated foster nestlings they raised). Thin gray lines represent the 95% confidence bands.

Naturally circulating male and female plasma T levels were measured with an EIA assay during the natural peak of T production. Plasma T levels varied within and between sexes, with males having higher levels (4.64 ± 0.39 ng/ml, range [1.76–10.55]) compared to females (0.97 ± 0.09 ng/ml, range [0.36–2.56]). Testosterone levels within brother and sister pairs positively co-varied (Fig. 1a; *r*_p_ = 0.41; F_1,37_ = 7.33; P = 0.010), indicating an intersexual correlation for T in our study population. Two data points seemed to represent overly high female T levels (Fig. 1a). However, these values fell fully within the relevant range of our study population, see^37^. Also, the variance distribution of the regression residuals were very close to normality (Shapiro-Wilk, W = 0.975; P = 0.54). Thus there are no empirical and statistical arguments to treat these values as outliers. Broad-sense heritability of T based on full-sibling comparisons was calculated as twice the intra-class correlation coefficient and is estimated at *h^2^* = 0.41 ± 0.31.

### Sexually antagonistic fitness variation

Offspring were cross-fostered at an early stage (day 0), to standardize parental work load across nests with all foster nests containing four unrelated nestlings (see methods). Average fledgling (day 20) body mass co-varied negatively between brothers and sisters (Fig. 1b; *r*_p_ = −0.45; F_1,22_ = 5.78; P = 0.025). The absolute mean difference between fledgling body mass raised by a brother and its sister was 8.0 ± 1.2% (range [0.6–23.0]).

### Intralocus sexual conflict

**Figure 2:**
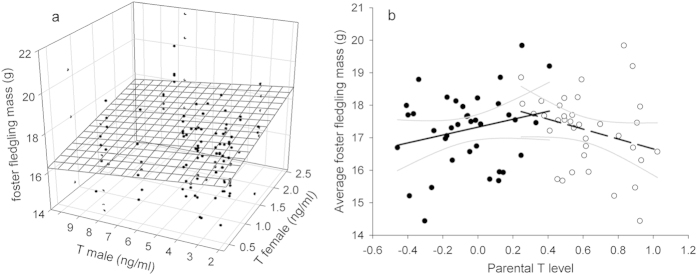
Sex-specific effects of plasma testosterone on parental fitness. Fledgling mass increases with testosterone levels of the foster mother, but decreases with testosterone levels of the foster father. Each dot in plot (**a**) represents individual fledgling mass in relation to the T level of both foster parents. Plot (**b**) represents average fledgling mass for a given foster nest in relation to both parents’ T level. Filled circles and the solid regression fit represent female parents. Open circles and the striped regression fit represent male parents. Thin gray lines represent the 95% confidence bands. T levels are log transformed for graphical clarity.

We explored the mechanism that drives this negative co-varying fitness estimate by relating fledging mass with T levels of the genetic and the foster parents. Fledgling body mass was not affected by the T levels of both genetic parents, not as an interaction (F_1,45_ = 0.60; P = 0.44), and neither as main effects (female testosterone level: F_1,45_ = 0.00; P = 0.99; male testosterone level: F_1,45_ = 0.08; P = 0.78). However, interestingly, effects of plasma T on fledging mass differed within broods between both foster parents (interaction female testosterone level by male testosterone level: F_1,54_ = 10.05; P = 0.003 [stepwise reduced model], F_1,45_ = 4.66; P = 0.0363 [full model], Fig. 2). Specifically, fledgling mass increased with increasing T levels of their foster mother (main effect: F_1,54_ = 14.6; P = 0.0003; effect size: *r*_p_ = 0.17), whereas the contrary was observed for foster fathers (main effect: F_1,54_ = 4.36; P = 0.041; *r*_p_ = −0.12; see Fig. 2a). When averaging foster fledgling mass within nests, it became clear that optimal male and female T levels lay close to each other (Fig. 2b), cf. opposite directional selection towards a state of monomorphism (see discussion). The average body mass of the genetic parents had no effect on fledgling mass variation (F_1,50_ = 2.91; P = 0.094). The random effects ‘genetic nest ID’ and ‘foster nest ID’ accounted respectively for 24.0% (likelihood ratio tests: χ^2^ = 8.0, P = 0.005) and 1.1% (χ^2^ = 0.0, P = 1.00) of the variance in the data. This indicates more similarities in fledgling body mass by decent, via an unknown common pre-hatching parental mechanism, than by the foster environment.

## Discussion

We aimed at gaining a better understanding of potential constraints for the evolution of phenotypic traits that are shared by both sexes. Specifically, we compared naturally circulating plasma T levels and an important aspect of fitness (here fledgling body mass) between parenting full-siblings. Such within-family approaches are powerful but are currently rather unexplored. We found that, within pairs of relatives, either a brother or his sisters raised offspring with high body mass. Such opposing fitness effects are indicative for sexual antagonistic selection during the period of parental care. However, these fitness differences were not driven by T, despite the fact that T had contrasting fitness effects for males and females, and despite an existing intersexual correlation of T levels.

Intersexual correlations between genetically related individuals are well-described for morphological traits like body size in a wide range of animal species (e.g. vertebrates^17^, insects^38^). Essentially, the same expectations hold for less stable phenotypic traits like behavioural^19^ or physiological traits^32^,^39^, but this is less well studied. We found significant positive co-variation in T levels of full-sib males and females. Our results within species concur with positive male-to-female T comparisons across a large number of fish^6^ and birds^4^,^8^,^9^, jointly suggesting that T levels are at least partly determined by the same set of genes in both sexes (although alternative explanations have been suggested^40^). Such intersexual genetic correlations may constrain independent male and female evolution and generate an intralocus sexual conflict if both sexes have different fitness optima for the focal trait. The latter will be thoroughly discussed further.

Studying intersexual correlations also allowed us to estimate heritable phenotypic trait variation. Surprisingly little is yet known about the heritability of T levels despite its significance for behavioural and evolutionary ecology^1^. Moreover, the limited number of studies are strongly biased towards mammals, especially humans^32^. Only two studies report fairly high heritability for maternally derived T levels in the egg yolk of Collared flycatchers (*Ficedula albicollis; h^2^* = 0.75 (0.23–1.26 CI)^41^ and Japanese quail (*Coturnix japonica; h^2^* = 0.42 ± 0.03SD)^42^. To the best of our knowledge, we here present some of the first empirical evidence on the heritability of peak plasma T levels in birds (*h^2^* = 0.41 ± 0.31). This outcome is based on full-sibling (brother-sister) comparisons and is generally in line with earlier reports, e.g. domestic pigs (*Sus domesticus*, *h^2^* *=* 0.37 ± 0.16^32^ and 0.38 ± 0.45^43^), bank voles (*Myodes glareolus*, *h^2^* ranges between 0.24 ± unknown SE and 0.58 ± 0.16) and red deer (*Cervus elaphus*, *h^2^* = 0.16 ± 0.06), overview in^32^. However, many of these heritability estimates should be treated with care, as the reported values may have been inflated as a result of environmental and maternal effects. Nevertheless, Mills *et al*.^44^ have recently shown a strong response to artificial selection for high and low individual T levels in bank voles. This resulted in a twofold difference in male T between both lines, even after one generation. This may further indicate that variation in plasma T levels has a significant genetic component, which makes it relevant to assess the potential occurrence of an intralocus sexual conflict.

Fledgling body mass negatively co-varied between brothers and sisters indicating opposing fitness patterns within pairs of relatives. Fledgling body mass is the result of accumulated amount of parental care and is believed to be a good predictor for recruitment probability^45^,^46^,^47^. Hence, if recruitment is high, so is the fitness of both parents. Here, we found that either a brother or his sister have a relatively high fitness, but not both. This sexually antagonistic variation during the period of parental care is an intriguing finding. It has important implications for both sexes, because a genotype that produces a male phenotype with relatively high fitness (e.g. good food providers with low T producing heavy fledglings) will, on average, produce a phenotype with lower fitness when expressed in a female (worse food providers with low T producing light fledglings). Similar observations have been made for reproductive success in adult fruit flies^48^, lifetime number of fledglings produced in great reed warblers^18^, lifetime number of recruits in collared flycatchers^36^ and lifetime number of offspring in red deer^35^. All the above antagonistic fitness patterns jointly indicate constrained evolution on causal male and female traits, while the mechanisms underlying such fitness patterns may differ. For example in great reed warblers (*Acrocephalus arundinaceus*), large ‘migratory’ and short ‘maneuverability’ wings are favoured in males and females, respectively, which largely explains the observed antagonistic fitness patterns^18^. We hypothesized that T is the causal phenotypic trait that drives our observed sexually antagonistic variation in fledging body mass, given (a) that T is a vital mediator of vertebrate life-history traits, (b) that T has marked sex-specific organizational and activational effects^21^, and (c) that the expression of several behavioural traits significant for parental investment are correlated with individual T levels^24^, see also^49^ for our model species. Importantly, based on our mid-parent - offspring covariation, fledgling body mass did not significantly relate to the body mass of the original parents, indicating a limited genetic component in this trait. Neither did T levels of the genetic parents explain variation in fledgling body mass, indicating e.g. restricted hormone-mediated maternal effects^50^,^51^. However, although genetic offspring share significant similarities by decent (via an undefined common mechanism, see random effects), the majority of the variation in fledgling body mass was explained by T levels of the foster parents.

The anticipated intralocus sexual conflict requires that optimal T-levels in males should be associated with low fitness in females (and vice versa)^15^,^16^. One of the clearest indications for this hypothesis comes again from the study on bank voles^44^, where female offspring from artificially selected low T lines had higher fitness when compared to female offspring from high T lines, while the opposite was true for male offspring. Likewise in the present study, we show sex-specific fitness effects of T. But intriguingly, these effects are in the opposite direction than expected. Specifically, fledgling body mass increased with T levels of the foster mother and decreased with T levels of the foster father. Males with high levels of T are generally more competitive in a context of sexual selection^21^. Indeed, T is of major importance for the expression of sexual traits and hence affects male fitness via intra- and inter-sexual competition. However, the advantage of high T levels is often traded-off against physiological costs and behavioural drawbacks (cf. challenge hypothesis^52^). The latter might well explain why fledgling mass, which relates to the stage of parental care, decreased with male T levels. Negative impact of male T on fledgling mass might, however, be offset by other advantages outside the period of parental care, e.g. via additional mating opportunities. In contrast, we observed a positive relationship between female fitness and T level, despite much lower variation in female T levels compared to males. This strongly contrasts with our expectations based on earlier research, indicating that elevated female T levels are generally associated with decreased parental investment^5^,^26^,^27^,^28^. However another study using canaries^49^ reported a similar positive effect of androgen levels on parental provisioning (measured as offspring growth rate), which was particularly strong in females. Although we argued earlier that T has a significant broad-sense heritability (41%), T levels may in part also be condition dependent. Indeed, Hinde *et al*.^49^ experimentally showed that high female androgen levels were associated with good food conditions. If so, female T levels likely signal general health conditions, which may ultimately result in better care and higher fledgling body mass. Although our experiment was performed under constant and equal conditions for all couples, this may be a potential mechanism explaining part of the T-related female fitness variation. However, further research is required to confirm this speculation.

Our observed fitness maxima for plasma T are much more similar than anticipated and rather indicate selection in favour of monomorphic male and female T levels, possibly because the intralocus sexual conflict may have been overcome. Indeed, our observations contrast the characterization of such an intralocus sexual conflict, which refers to the situation where males and females have clear distinct fitness optima for a given trait at the same loci^53^. Further empirical support for sexual conflict resolution comes from a recent micro-array study with dark-eyed junco’s (*Junco hyemalis*), showing sex-differences in gene expression of two brain regions (medial amygdala and hypothalamus) following T administration^31^. Also the histological research by Gahr^54^ demonstrated that although T-regulated song behaviour is expressed in male and female forest weavers (*Ploceus bicolor*), gene expression within the vocal control brain areas was clearly sexually dimorphic. These studies indicate the potential to regulate at least some T-related processes independently in both sexes. Indeed, sex-biased gene expression can evolve rapidly when driven by sexual selection and sexual conflict^55^, ultimately resulting in present-day genetic conflict resolution.

However, we still observed a clear sexual dimorphism in peak T levels in our study system, with males having on average almost five times higher values than females. Sexual dimorphism in a shared trait is further considered as an indication for sexual conflict resolution^14^,^15^,^16^. From a mechanistic point of view, this particular sexual dimorphism likely relates to the main tissue for T production, which differs between males (testes) and females (ovaries). This distinct origin may not only result in a different magnitude of plasma T between sexes, but may also have important additional effects for the duration of T production^1^. Indeed, plasma T levels in several European and North-American passerine birds rise at the beginning of the breeding season towards a peak near egg laying and decrease again to baseline levels during the period of offspring provisioning, with a longer and higher peak in males compared to females^4^,^33^,^56^,^57^. We nevertheless demonstrate sex-specific effects of peak T levels on fledgling mass, despite a time lag between both quantified parameters. Testosterone levels may be correlated within individuals across successive stages of reproduction, but this remains speculative. In fact, we are aware of only one study^33^ that measured natural (i.e. not experimentally increased) levels of plasma T at different points in time over the reproductive season on an individual basis. This study revealed that dominant individuals had higher T levels than subordinates during territorial establishment, as well as when nestlings were reared, suggesting correlated within-individual T levels across successive stages of reproduction. However, for future research we suggest to assess within-individual behavioural and reproductive trade-offs driven by temporal dynamics of T in both sexes (see also^1^). Such data is notoriously difficult to gather, particularly in the wild, but it remains one of the future challenges. Additionally, the period of elevated T may vary among individuals, which could reflect variation in life-history strategies^58^. For example, males with longer periods of T may start breeding earlier, breed more often during a single season or realize more extra-pair fertilizations.

To conclude, our study contributes to a rapidly developing research field within evolutionary biology about the extent to which males and females constrain each other’s evolution^7^,^14^,^15^,^59^. Studies comparing phenotypic and/or reproductive traits among relatives, like our comparative full-sibling approach, are rather rare. However, similar techniques become increasingly popular along with the wider applicability of animal models^18^,^60^. The study of genomic conflicts is at its infancy, but many important questions remain to be answered, e.g. related to the speed of conflict resolution, coevolution of suites of traits and its role in speciation; reviewed in^7^,^14^,^15^. Studies across a variety of taxa involving related family members are therefore key to understand genomic conflicts and their resolution. Whether and how intersexual genetic correlations prevent both sexes to reach their fitness optimum for plasma T levels is furthermore subject to an intense debate among evolutionary biologists^9^,^10^,^11^,^12^. On the one hand, we indeed found evidence for constrained evolution supported by positive intersexual correlations for T levels and T having opposing effects on male and female fitness (i.e. fledgling body mass). On the other hand, T levels showed clear sexual dimorphism and were not convincingly subject to an intralocus sexual conflict, indicating significant functional and/or mechanistic differences between both sexes. We expect independent male and female evolution of T levels, only within the limits set by the intersexual genetic correlation of this trait. Consequently, our results suggest that both extreme views (constrained vs. independent evolution) should be integrated to understand the contemporary expression of quantitative traits, including the level of circulating male and female plasma T. This has further important implications for understanding the expression of numerous morphological, physiological and behavioural traits^2^,^30^, that are mediated by these circulating T levels. Furthermore, plasma T levels reflect only one part of the entire T machinery, for which many loci are involved for production, transport and/or receptor binding of T. This further supports the idea that both sexes inevitably affect one another’s evolution. However, we would like to stress that temporal dynamics in the regulation of T in males and females may play a central role in the process of conflict resolution for all these complex intertwined aspects, and more research on this is urgently needed. Finally, it appeared unlikely that plasma T is part of the proximate mechanisms driving sexually antagonistic selection on reproductive success in our study species. Thus the underlying mechanisms of this antagonistic fitness pattern yet remain obscure.

## Methods

### Study species and animal keeping

The methods were carried out in accordance with the approved guidelines by the Ethical Committee of the University of Antwerp (ID: 2014–72). We used a total of 80 adult Fife Fancy canaries from our own outbred pedigreed population. Specifically, the dataset consisted of 40 full-sibling pairs, i.e. two (N = 15) or three (N = 25) year old brother and sister duos originating from the same genetic parents. These relatives breed in separate cages to avoid inbreeding depression. Five weeks prior to the breeding period, males and females were housed separately in two large indoor aviaries. All birds experienced a long light regime during this period (14:10, L:D) and had access to seeds and water *ad libitum*. Egg food was provided twice a week. After five weeks of long light regime, males were housed in individual cages (50 × 64 × 40 cm, GEHU cages, The Netherlands) for three weeks to mimic territorial establishment. Subsequently, each male was paired by allocating an unrelated female to the cage and nesting materials were provided. Progress on nest building, egg laying and incubation was monitored daily^61^. A successful clutch with at least one egg (mean clutch size = 3.97 ± 0.17 eggs) was produced in 37 nests. Hatching was synchronized within broods to minimize sibling competition and to facilitate cross-fostering^49^,^62^. Enriched egg food was provided on a daily basis after hatching (day 0). Nestlings were cross-fostered at an early stage (day 0), to standardize parental work load across nests with all foster nests containing four unrelated nestlings. Nestlings were cross-fostered with respect to nestling age (max 12 h difference between nestlings) to minimize within-nest competition and with respect to egg order (nestlings hatched from eggs with different egg order) to minimize inflation of maternal effects. Nestlings were individually marked with a unique within-nest color on their back, using non-toxic pens (Artline®70N) which was reapplied when necessary. They were ringed with a unique code on day 7. A total of 30 nests successfully raised offspring until fledging. Fitness comparisons (see further) were possible for less (n = 24) sibling pairs, because nest failure does not further allow comparison between the failed parents and their respective brother and sister.

### Plasma testosterone measurements

Blood (±100 μl) was sampled from the alar wing vein for measuring plasma T levels near the expected seasonal peak in T concentrations^4^,^33^,^52^,^56^,^57^. A major advantage of comparing natural circulating T levels is the avoidance of potentially confounding effects of hormone manipulations^9^. For males, blood was sampled in the period before couples were made, which mimics the period of territorial establishment. For females this was done on the first day of egg laying. Furthermore, blood was always sampled between 10 am and 1 pm to reduce potential diurnal variation in T levels^1^. Plasma concentrations of T were measured using an enzyme-linked immunoassay EIA kit (Enzo Life Sciences, ADI-900-065). Sensitivity of the assay is 5.67 pg/ml and shows 100% cross-reactivity with testosterone, 14.63% with 19-hydroxytestosterone, 7.2% with androstendione and <1% with other steroid compounds. A subset of samples were analyzed twice with and without diethyl ether extraction, which gave nearly identical outcomes (Pearson correlation: r_p_ = 0.97, β = 1.03, P < 0.0001, N = 14–6 females and 8 males) and there were no indications for different effects for both sexes. Hence lab-analyses were performed on non-extracted samples. The assay kit was validated by ensuring parallelism of serial dilutions of plasma samples with the standard 4-parameter logistic curve. Samples were diluted 1:15 into the supplied assay buffer and concentrations of T were assessed with the standard curve for each assay separately. Repeatability was calculated to determine the variation in the data caused by measurement errors at both levels, within and across 96-well assays (N = 10 assays) and was calculated as the proportion of the variance between individuals to the total variance, i.e. between and within individual^63^. Each plasma sample followed the entire protocol in duplo, resulting in a within-assay repeatability of 99.1% (N = 426). Furthermore, a set of randomly chosen samples were measured twice in different assays. Across-assay repeatability was 96.8% (N = 39), indicating overall limited measurement error. Finally, blood was sampled twice to assess repeatability in a parallel study. We left ten days between sampling events for males, while females were sampled on the first day of egg laying in the first and second clutch (35 ± 1 days in between). Repeatability between both sample events was calculated as in Dingemanse and Dochtermann^63^ and was significant for males (0.36 ± 0.16, χ^2^_1_ = 6.3; P = 0.012), females (0.31 ± 0.12, χ^2^_1_ = 4.9; P = 0.027) and both sexes combined (0.34 ± 0.11, χ^2^_1_ = 10.8; P = 0.001). Means of both measurements were used for further analyses and are considered a more accurate estimate of individual T. Our reported mean T values in males (4.6 ± 0.4 ng/ml) and females (1.0 ± 0.1 ng/ml) fall well within the range of previously reported values for our population, measured at similar life-history stages, but with radioimmunoassay (males: 5.6 ± 0.6 ng/ml^64^, 5.9 ± 1.0 ng/ml^65^ and females: 0.8 ± 0.1–1.1 ± 0.1 ng/ml^37^). However, we note that care is needed when comparing absolute values across studies^34^.

### Quantifying parental fitness

When the offspring reached an age of 20 days (±1day), the female started to prepare the nest for the next clutch. This corresponds with the time that the nestlings fledge. Fledgling body mass was measured at day 20 and is considered to represent a fitness estimate. Our cross-foster design averaged genetic and pre-natal maternal effects, so our fitness estimate likely forms a reliable indicator of parental quality. In other words, the fitness estimate indicates a parent’s capability to produce heavy offspring, i.e. with high nutritional reserves^66^ and resulting high survivorship and recruiting probabilities^45^,^47^,^67^, in standardized foster nests. In great tits (*Parus major*) for example, a 10% increase in fledgling body mass can result in a 30% higher recruitment probability^45^. Hence, if recruitment is high, so is the fitness of both parents. In the current study, we focus on this single fitness estimate, although we acknowledge that an individual’s fitness is also determined by T-related intra- and inter-sexual competition outside this period. The results will be discussed accordingly.

### Statistical procedure

The first part of the analyses involved full-sibling (brother/sister) comparisons of peak plasma T levels (assessing intersexual correlations) and average fledgling mass as parental fitness estimate (assessing sexual antagonistic selection). Separate linear regression models were performed in which a given male trait was regressed against its sister’s trait. In addition, we measured the broad-sense heritability (*h^2^*) of T in full-siblings by multiplying the intraclass correlation coefficient by two, as r = 0.5 between full siblings^68^. Specifically, the intraclass correlation coefficient was the variance within sibling pairs divided by the total (i.e. within- and between sibling pairs) variance, using a univariate mixed model with family (sibling pair) as a random effect^63^. Given the strong sexual dimorphism in T levels in our dataset (see results), variance calculated within sibling pairs is largely inflated. Individual deviation from the sex-specific population mean was used to standardize for this sexual difference. This way, the variance within the sexes remained the same, but T levels could be more reliably compared between brothers and sisters.

In a second part of the analyses we explored the mechanism that drives signs of sexually antagonistic selection by relating fledgling mass with sex-specific T levels. By doing so, we tested the potential occurrence of an intralocus sexual conflict. A linear mixed model (LMM) was used in which variation in individual fledgling body mass was explained by (1) T level of both the foster father and foster mother, as well as their interaction. Similarly (2), we included T levels of the genetic parents to assess any parental, and particularly maternal, effect on offspring body mass^49^,^50^. Testosterone levels were not related between parents within nests (r_p_ = 0.08; N = 36; P = 0.65). Furthermore (3), our cross-foster design generated standardized nests in which genetic and maternal effects were equalized as much as possible among the foster nestlings. Nonetheless, average body mass of the original parents was added to the model to adjust for potential genetic effects in this trait. In addition (4), given the genetic similarities among nestlings within the original nest before cross-fostering and the similar environmental conditions within each foster nest, both genetic nest ID and foster nest ID were added as independent random factors to the model to adjust for a bias in statistical independence. This mixed model was reduced using backward stepwise elimination of the least significant (highest P value) fixed effects, until only significant (P ≤ 0.05) variables remained (unless a main effect is part of a significant higher order interaction).

Assumptions for normal distribution were always met (Shapiro-Wilk: W ≥ 0.96) based on the residuals in each of the above analyses. All statistical tests are performed in SAS 9.3 (SAS Institute Inc., Cary, NC, U.S.A.) and are two-tailed with an alpha significance level of 0.05. Mean values ± 1 S.E. are mentioned throughout the text, unless otherwise mentioned.

## Additional Information

**How to cite this article**: Iserbyt, A. *et al*. Sexually antagonistic selection during parental care is not generated by a testosterone-related intralocus sexual conflict – insights from full-sib comparisons. *Sci. Rep*. **5**, 17715; doi: 10.1038/srep17715 (2015).

## References

[b1] Kempenaers B., Peters A. & Foerster K. Sources of individual variation in plasma testosterone levels. Philos. Trans. R. Soc. B-Biol. Sci. 363, 1711–1723 (2008). 10.1098/rstb.2007.0001 PMC2367619 18048297

[b2] Hau M. Regulation of male traits by testosterone: implications for the evolution of vertebrate life histories. BioEssays 29, 133–144 (2007). 17226801 10.1002/bies.20524

[b3] Staub N. L. & De Beer M. The role of androgens in female vertebrates. Gen. Comp. Endocrinol. 108, 1–24 (1997). 9378263 10.1006/gcen.1997.6962

[b4] Ketterson E. D., Nolan V. & Sandell M. Testosterone in females: mediator of adaptive traits, constraint on sexual dimorphism, or both? Am. Nat. 166, S85–S98 (2005). 16224714 10.1086/444602

[b5] Veiga J. P. & Polo V. Fitness consequences of increased testosterone levels in female spotless starlings. Am. Nat. 172, 42–53 (2008). 18532881 10.1086/587850

[b6] Mank J. E. The evolution of sexually selected traits and antagonistic androgen expression in Actinopterygiian Fishes. Am. Nat. 169, 142–149 (2007). 17206593 10.1086/510103

[b7] Mokkonen M. & Crespi B. J. Genomic conflicts and sexual antagonism in human health: insights from oxytocin and testosterone. Evol. Appl. 8, 307–325 (2015). 25926877 10.1111/eva.12244 PMC4408143

[b8] Møller A. P., Garamszegi L. Z., Gil D., Hurtrez-Boussès S. & Eens M. Correlated evolution of male and female testosterone profiles in birds and its consequences. Behav. Ecol. Sociobiol. 58, 534–544 (2005).

[b9] Goymann W. & Wingfield J. C. Male-to-female testosterone ratios, dimorphism, and life history–what does it really tell us? Behav. Ecol. 25, 685–699 (2014).

[b10] Groothuis T. G. G., de Jong B. & Müller M. In search for a theory of testosterone in female birds: a comment on Goymann and Wingfield. Behav. Ecol. 25, 702–704 (2014).

[b11] Ketterson E. D. Male and female testosterone-is one sex made in the image of the other? A comment on Goymann and Wingfield. Behav. Ecol. 25, 702–702 (2014).

[b12] Garamszegi L. Z. Female peak testosterone levels in birds tell an evolutionary story: a comment on Goymann and Wingfield. Behav. Ecol. 25, 700–701 (2014).

[b13] Lande R. Sexual dimorphism, sexual selection, and adaptation in polygenic characters. Evolution. 34, 292–305 (1980). 10.1111/j.1558-5646.1980.tb04817.x 28563426

[b14] Bonduriansky R. & Chenoweth S. F. Intralocus sexual conflict. Trends Ecol. Evol. 24, 280–288 (2009). 19307043 10.1016/j.tree.2008.12.005

[b15] Van Doorn G. S. Intralocus sexual conflict. Ann. N. Y. Acad. Sci. 1168, 52–71 (2009). 19566703 10.1111/j.1749-6632.2009.04573.x

[b16] Cox R. M. & Calsbeek R. Sexually antagonistic selection, sexual dimorphism, and the resolution of intralocus sexual conflict. Am. Nat. 173, 176–187 (2009). 19138156 10.1086/595841

[b17] Merilä J., Sheldon B. C. & Ellegren H. Quantitative genetics of sexual size dimorphism in the collared flycatcher, Ficedula albicollis. Evolution. 52, 870–876 (1998). 10.1111/j.1558-5646.1998.tb03711.x 28565242

[b18] Tarka M., Akesson M., Hasselquist D. & Hansson B. Intralocus sexual conflict over wing length in a wild migratory bird. Am. Nat. 183, 62–73 (2014). 24334736 10.1086/674072

[b19] Long T. A. F. & Rice W. R. Adult locomotory activity mediates intralocus sexual conflict in a laboratory-adapted population of *Drosophila melanogaster*. Proc. R. Soc. B - Biol. Sci. 274, 3105–3112 (2007). 10.1098/rspb.2007.1140 PMC2293944 17925279

[b20] Svensson E. I., McAdam A. G. & Sinervo B. Intralocus sexual conflict over immune defense, gender load, and sex-specific signaling in a natural lizard population. Evolution. 63, 3124–3135 (2009). 19624721 10.1111/j.1558-5646.2009.00782.x

[b21] Adkins-Regan E. in Hormones and Animal Social Behaviour 1st edn. (Princeton University Press, 2005).

[b22] Owen-Ashley N. T., Hasselquist D. & Wingfield J. C. Androgens and the immunocompetence handicap hypothesis: unraveling direct and indirect pathways of immunosuppression in song sparrows. Am. Nat. 164, 490–505 (2004). 15459880 10.1086/423714

[b23] Alonso-Alvarez C., Bertrand S., Faivre B., Chastel O. & Sorci G. Testosterone and oxidative stress: the oxidation handicap hypothesis. Proc. R. Soc. B-Biol. Sci. 274, 819–825 (2007). 10.1098/rspb.2006.3764 PMC2093982 17251089

[b24] Lynn S. E. Behavioral insensitivity to testosterone: why and how does testosterone alter paternal and aggressive behavior in some avian species but not others? Gen. Comp. Endocrinol. 157, 233–240 (2008). 18579140 10.1016/j.ygcen.2008.05.009

[b25] Von Engelhardt N. & Groothuis T. G. G. in Horm. Reprod. Vertebr 1st edn. (Noris D. O. & Lopez K. H.) 91–127 (Elsevier Inc., 2011).

[b26] Gerlach N. M. & Ketterson E. D. Experimental elevation of testosterone lowers fitness in female dark-eyed juncos. Horm. Behav. 63, 782–790 (2013). 23523740 10.1016/j.yhbeh.2013.03.005

[b27] O’Neal D. M., Reichard D. G., Pavilis K. & Ketterson E. D. Experimentally-elevated testosterone, female parental care, and reproductive success in a songbird, the Dark-eyed Junco (*Junco hyemalis*). Horm. Behav. 54, 571–578 (2008). 18585386 10.1016/j.yhbeh.2008.05.017

[b28] Lahaye S. E. P., Eens M., Darras V. M. & Pinxten R. Male-like testosterone levels inhibit oviposition in a female parrot: A breeding experiment in budgerigars. Curr. Zool. 61, 586–595 (2015).

[b29] Abbott J. K., Innocenti P., Chippindale A. K. & Morrow E. H. Epigenetics and sex-specific fitness: an experimental test using male-limited evolution in *Drosophila melanogaster*. PLoS One 8, e70493 (2013). 23922998 10.1371/journal.pone.0070493 PMC3726629

[b30] McGlothlin J. W. & Ketterson E. D. Hormone-mediated suites as adaptations and evolutionary constraints. Philos. Trans. R. Soc. Lond. B. Biol. Sci. 363, 1611–1620 (2008). 18048296 10.1098/rstb.2007.0002 PMC2606720

[b31] Peterson M. P. . Testosterone affects neural gene expression differently in male and female juncos: a role for hormones in mediating sexual dimorphism and conflict. PLoS One 8, e61784 (2013). 23613935 10.1371/journal.pone.0061784 PMC3627916

[b32] Pavitt A. T., Walling C. A., Pemberton J. M. & Kruuk L. E. B. Heritability and cross-sex genetic correlations of early-life circulating testosterone levels in a wild mammal. Biol. Lett. 10, 20140685 (2014). 25428929 10.1098/rsbl.2014.0685 PMC4261863

[b33] Parisot M. . Social competition and plasma testosterone profile in domesticated canaries: An experimental test of the challenge hypothesis. Horm. Behav. 48, 225–232 (2005). 15878578 10.1016/j.yhbeh.2005.02.011

[b34] Garamszegi L. Z., Eens M. & Hurtrez-Boussès S. & Møller, a. P. Testosterone, testes size, and mating success in birds: A comparative study. Horm. Behav. 47, 389–409 (2005). 15777805 10.1016/j.yhbeh.2004.11.008

[b35] Foerster K. . Sexually antagonistic genetic variation for fitness in red deer. Nature 447, 1107–1110 (2007). 17597758 10.1038/nature05912

[b36] Brommer J. E., Kirkpatrick M., Qvarnström A. & Gustafsson L. The intersexual genetic correlation for lifetime fitness in the wild and its implications for sexual selection. PLoS One 2, e744 (2007). 17710144 10.1371/journal.pone.0000744 PMC1939732

[b37] Müller W., Groothuis T. G. G., Goerlich V. C. & Eens M. GnRH - a missing link between testosterone concentrations in yolk and plasma and its intergenerational effects. PLoS One 6, e22675 (2011). 21829476 10.1371/journal.pone.0022675 PMC3145665

[b38] Abbott J. K. & Svensson E. I. Morph-specific variation in intersexual genetic correlations in an intra-specific mimicry system. Evol. Ecol. Res. 12, 105–118 (2010).

[b39] Schroderus E. . Intra- and intersexual trade-offs between testosterone and immune system: Implications for sexual and sexually antagonistic selection. Am. Nat. 176, E90–E97 (2010). 20712516 10.1086/656264

[b40] Goymann W. & Wingfield J. C. Correlated evolution of female and male testosterone--internal constraints or external determinants? A response to comments on Goymann and Wingfield. Behav. Ecol. 25, 704–705 (2014).

[b41] Tschirren B., Sendecka J., Groothuis T. G. G., Gustafsson L. & Doligez B. Heritable variation in maternal yolk hormone transfer in a wild bird population. Am. Nat. 174, 557–564 (2009). 19737108 10.1086/605379

[b42] Okuliarova M., Groothuis T. G. G., Skrobánek P. & Zeman M. Experimental evidence for genetic heritability of maternal hormone transfer to offspring. Am. Nat. 177, 824–834 (2011). 21597258 10.1086/659996

[b43] Bates R. O. . Genetic parameter estimates for reproductive traits of male and female littermate swine. J. Anim. Sci. 63, 377–385 (1986). 3531134 10.2527/jas1986.632377x

[b44] Mills S. C., Koskela E. & Mappes T. Intralocus sexual conflict for fitness: sexually antagonistic alleles for testosterone. Proc. R. Soc. B-Biol. Sci. 279, 1889–1895 (2012). 10.1098/rspb.2011.2340 PMC3311893 22171083

[b45] Both C., Visser M. E. & Verboven N. Density-dependent recruitment rates in great tits: the importance of being heavier. Proc. R. Soc. B-Biol. Sci. 266, 465–469 (1999).

[b46] Bowers E. K., Nietz D., Thompson C. F. & Sakaluk S. K. Parental provisioning in house wrens: effects of varying brood size and consequences for offspring. Behav. Ecol. 25, 1485–1493 (2014).

[b47] Maness T. J. & Anderson D. J. Predictors of juvenile survival in birds. Ornithol. Monogr. 78, 1–55 (2013).

[b48] Chippindale A. K., Gibson J. R. & Rice W. R. Negative genetic correlation for adult fitness between sexes reveals ontogenetic conflict in Drosophila. Proc. Natl. Acad. Sci. 98, 1671–1675 (2001). 11172009 10.1073/pnas.041378098 PMC29315

[b49] Hinde C. A., Buchanan K. L. & Kilner R. M. Prenatal environmental effects match offspring begging to parental provisioning. Proc. R. Soc. B-Biol. Sci. 276, 2787–2794 (2009). 10.1098/rspb.2009.0375 PMC2839951 19419982

[b50] Schwabl H. Maternal testosterone in the avian egg enhances postnatal growth. Comp. Biochem. Physiol. 114, 271–276 (1996). 10.1016/0300-9629(96)00009-6 8759148

[b51] Groothuis T. G. G., Müller W., von Engelhardt N., Carere C. & Eising C. Maternal hormones as a tool to adjust offspring phenotype in avian species. Neurosci. Biobehav. Rev. 29, 329–352 (2005). 15811503 10.1016/j.neubiorev.2004.12.002

[b52] Wingfield J. C., Hegner R. E., Dufty A. M. & Ball G. F. The ‘Challenge Hypothesis’: Theoretical implications for patterns of testosterone secretion, mating systems, and breeding strategies. Am. Nat. 136, 829–846 (1990).

[b53] Rice W. R. & Chippindale A. K. Intersexual ontogenetic conflict. J. Evol. Biol. 14, 685–693 (2001).

[b54] Gahr M., Metzdorf R., Schmidl D. & Wickler W. Bi-directional sexual dimorphisms of the song control nucleus HVC in a songbird with unison song. PLoS One 3, e3073 (2008). 18728787 10.1371/journal.pone.0003073 PMC2518117

[b55] Harrison P. W. . Sexual selection drives evolution and rapid turnover of male gene expression. Proc. Natl. Acad. Sci. 112, 4393–4398 (2015). 25831521 10.1073/pnas.1501339112 PMC4394296

[b56] Johnsen T. S. Behavioural correlates of testosterone and seasonal changes of steroids in red-winged blackbirds. Anim. Behav. 55, 957–965 (1998). 9632481 10.1006/anbe.1997.0642

[b57] Goymann W., Landys M. M. & Wingfield J. C. Distinguishing seasonal androgen responses from male-male androgen responsiveness–Revisiting the Challenge Hypothesis. Horm. Behav. 51, 463–476 (2007). 17320880 10.1016/j.yhbeh.2007.01.007

[b58] Raouf S. A., Parker P. G., Ketterson, E. D., Jr, V. N. & Ziegenfus, C. Testosterone affects reproductive success by influencing extra-pair fertilizations in male dark-eyed juncos (Aves: Junco hyemalis). Proc. R. Soc. B Biol. Sci. 264, 1599–1603 (1997).

[b59] Pennell T. M. & Morrow E. H. Two sexes, one genome: the evolutionary dynamics of intralocus sexual conflict. Ecol. Evol. 3, 1819–1834 (2013). 23789088 10.1002/ece3.540 PMC3686212

[b60] Wilson A. J. . An ecologist’s guide to the animal model. J. Anim. Ecol. 79, 13–26 (2010). 20409158 10.1111/j.1365-2656.2009.01639.x

[b61] Iserbyt A., Farrell S., Eens M. & Müller W. Sex-specific negotiation rules in a costly conflict over parental care. Anim. Behav. 100, 52–58 (2015).

[b62] Estramil N., Eens M. & Müller W. Coadaptation of offspring begging and parental provisioning - an evolutionary ecological perspective on avian family life. PLoS One 8, e70463 (2013). 23894662 10.1371/journal.pone.0070463 PMC3716698

[b63] Dingemanse N. J. & Dochtermann N. A. Quantifying individual variation in behaviour: mixed-effect modelling approaches. J. Anim. Ecol. 82, 39–54 (2013). 23171297 10.1111/1365-2656.12013

[b64] Vergauwen J., Groothuis T. G. G., Eens M. & Müller W. Testosterone influences song behaviour and social dominance - but independent of prenatal yolk testosterone exposure. Gen. Comp. Endocrinol. 195, 80–87 (2014). 24211320 10.1016/j.ygcen.2013.10.014

[b65] Müller W., Heylen D., Eens M., Rivera-Gutierrez H. F. & Groothuis T. G. G. An experimental study on the causal relationships between (ecto-)parasites, testosterone and sexual signalling. Behav. Ecol. Sociobiol. 67, 1791–1798 (2013).

[b66] Peig J. & Green A. J. New perspectives for estimating body condition from mass/length data: the scaled mass index as an alternative method. Oikos 118, 1883–1891 (2009).

[b67] Bowers E. K. . Neonatal body condition, immune responsiveness, and hematocrit predict longevity in a wild bird population. Ecology 95, 3027–3034 (2014). 25505800 10.1890/14-0418.1 PMC4260523

[b68] Boake C. R. B. Quantitative genetic studies of behavioral evolution. Quant. Genet. Stud. Behav. Evol. (The University of Chicago Press, 1994).

